# Fully automated pipeline for detection of sex linked genes using RNA-Seq data

**DOI:** 10.1186/s12859-015-0509-0

**Published:** 2015-03-11

**Authors:** Monika Michalovova, Zdenek Kubat, Roman Hobza, Boris Vyskot, Eduard Kejnovsky

**Affiliations:** Department of Plant Developmental Genetics, Institute of Biophysics, Academy of Sciences of the Czech Republic, Kralovopolska 135, CZ-61200 Brno, Czech Republic; Centre of the Region Hana for Biotechnological and Agricultural Research, Institute of Experimental Botany, Slechtitelu 31, 78371 Olomouc, Czech Republic; Current address: Department of Biology, Pennsylvania State University, University Park, PA 16802 USA

**Keywords:** Sex chromosomes, RNA-Seq, Segregation analysis, *Rumex acetosa*

## Abstract

**Background:**

Sex chromosomes present a genomic region which to some extent, differs between the genders of a single species. Reliable high-throughput methods for detection of sex chromosomes specific markers are needed, especially in species where genome information is limited. Next generation sequencing (NGS) opens the door for identification of unique sequences or searching for nucleotide polymorphisms between datasets. A combination of classical genetic segregation analysis along with RNA-Seq data can present an ideal tool to map and identify sex chromosome-specific expressed markers. To address this challenge, we established genetic cross of dioecious plant *Rumex acetosa* and generated RNA-Seq data from both parental generation and male and female offspring.

**Results:**

We present a pipeline for detection of sex linked genes based on nucleotide polymorphism analysis. In our approach, tracking of nucleotide polymorphisms is carried out using a cross of preferably distant populations. For this reason, only 4 datasets are needed – reads from high-throughput sequencing platforms for parent generation (mother and father) and F1 generation (male and female progeny). Our pipeline uses custom scripts together with external assembly, mapping and variant calling software. Given the resource-intensive nature of the computation, servers with high capacity are a requirement. Therefore, in order to keep this pipeline easily accessible and reproducible, we implemented it in Galaxy – an open, web-based platform for data-intensive biomedical research. Our tools are present in the Galaxy Tool Shed, from which they can be installed to any local Galaxy instance. As an output of the pipeline, user gets a FASTA file with candidate transcriptionally active sex-linked genes, sorted by their relevance. At the same time, a BAM file with identified genes and alignment of reads is also provided. Thus, polymorphisms following segregation pattern can be easily visualized, which significantly enhances primer design and subsequent steps of wet-lab verification.

**Conclusions:**

Our pipeline presents a simple and freely accessible software tool for identification of sex chromosome linked genes in species without an existing reference genome. Based on combination of genetic crosses and RNA-Seq data, we have designed a high-throughput, cost-effective approach for a broad community of scientists focused on sex chromosome structure and evolution.

**Electronic supplementary material:**

The online version of this article (doi:10.1186/s12859-015-0509-0) contains supplementary material, which is available to authorized users.

## Background

Sex chromosomes represent a unique “chromosomal elements” appearing in most gonochoristic animals and some plant dioecious species [1]. Since most living organisms are still far from being sequenced, mining of data about structure and function of individual sex chromosomes remains a largely unaddressed issue. Various molecular methods have been employed to identify sex specific markers and genes. In these approaches, DNA from individual sex chromosome is either mechanically separated by microdissection or flow-sorting of sex chromosomes [2], followed by construction of libraries and sequencing [3], or alternatively, sex-linked markers have been isolated by various molecular methods such as Restriction Fragment Length Polymorphism (RFLP), Amplified Fragment Length Polymorphism (AFLP) or Representational Difference Analysis (RDA) [4].

Next generation sequencing has become a unique platform for large-scale analysis of genome structure and dynamics. The use of massive sequencing data has broadly been adopted in genome and transcriptome comparative studies, for characterization of species and/or tissue specific expression profiles, identification of disease associated genes, and comparative genomics analysis [5].

In principle, RNA-Seq data from each individual of a cross can be processed separately or, alternatively, progeny data can be pooled (males and females separately) to minimize sequencing costs and extent of computation.

Here, we present a fully automated pipeline for detection of sex-linked genes that combines classical genetic segregation analysis with RNA-Seq data for male and female parent generation and pooled male and female progeny. This pipeline has been used for the identification of X- and Y-linked genes in *Rumex acetosa* (sorrel), a model dioecious plant species with XX/XY_1_Y_2_ sex chromosome system. The generated data were verified using PCR with newly identified sex-specific primers.

### Biological material and patterns of segregation used by our algorithm

**Figure 1:**
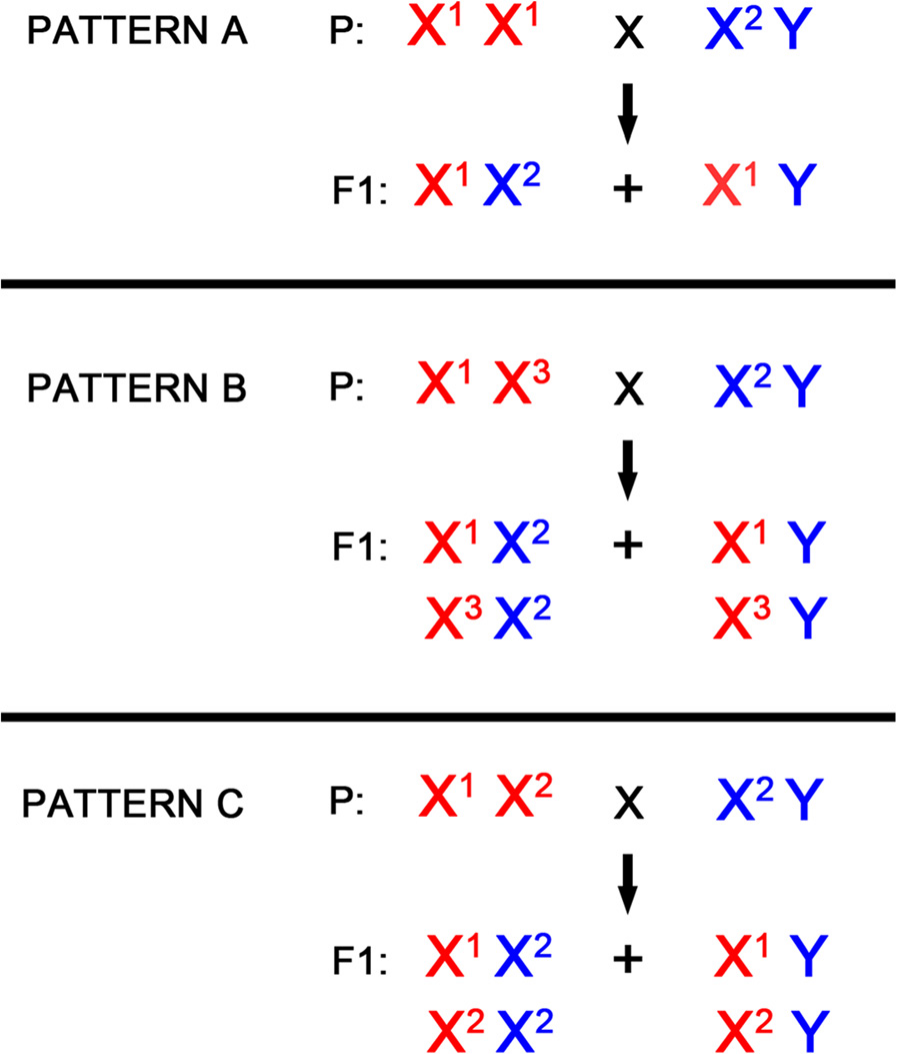
**Segregation patterns.** All possible combinations of sex-linked alleles in parent generation are listed. Alleles on the X chromosome exhibit criss-cross inheritance, while alleles on the Y chromosome can only be transmitted from father to son (holandric inheritance). Segregation patterns are used as the starting point for the design of filtering rules.

Possible patterns of sex gene segregation are shown in Figure 1. In pattern A, the mother is homozygous X^1^X^1^ (both alleles/variants of the gene are identical), while the father has two different alleles (X^2^Y). Thus, all sons possess the same allele variant as the mother - X^1^, and Y allele inherited from the father. All daughters inherit one allele from each parent, resulting in the X^1^X^2^ genotype. In pattern B, the mother is heterozygous X^1^X^3^ while the father has a different X linked variant (X^2^). According to segregation rules, the sons will be X^1^Y or X^3^Y and daughters X^1^X^2^ or X^3^X^2^ (all daughters will possess X^2^ from father). In pattern C, the mother is heterozygous and shares one allele (X^2^) with the father.

## Implementation

**Figure 2:**
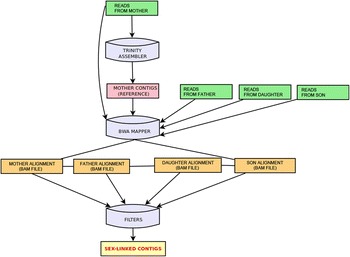
**Simplified workflow.** Mother’s reads are assembled with Trinity assembler and newly created contigs are then used as a reference to which all reads are mapped. This yields into 4 bam files storing alignments. After applying filters, X-linked contigs are identified. For detection of Y-linked genes, reference contigs are assembled from male reads (together male parent and male progeny)

Workflow of the pipeline used in the present study is visualized in Figure 2. The feasibility of our approach was tested using the cross of male and female individuals of *R. acetosa*. The total RNA was isolated from young leaves [6] and oligo dT primers were used in order to enrich for polyA RNA. Two very distant lineages were chosen for the study - mother lineage being *R. acetosa*, Almería, Spain while the father lineage was *R. acetosa*, Brno-Reckovice, Czech Republic. 5 male and 5 female individuals from F1 generation were chosen for separate isolation, test of integrity and concentration measurements. Finally, male and female RNA was pooled in the same proportions (1:1:1:1:1) and sequenced along with mother and father samples on Illumina HiSeq with resulting paired-end reads displaying a length peak of 87 bp for both forward and reverse reads. With an estimate of 2% of the *R. acetosa* genome being transcribed, this leads us to an approximation of 62x coverage for the assembly of reference X-linked genes and 44x coverage for the assembly of reference Y-linked genes [7].

The pipeline therefore requires four datasets with preprocessed RNA-Seq reads (adaptors must be removed and reads must be gently trimmed on quality; all these tasks can be performed using Galaxy) - mother reads, father reads, reads of pooled female progeny and reads of pooled male progeny. Both, FASTA and FASTQ files are accepted and can be easily interchangeable in Galaxy workflow.

For maximal efficiency of our pipeline, we recommend the following guidelines:

1. Reads from the mother should be the most abundant since they are essential for deriving X-linked contigs,
2. Attention should be paid such that even numbers of individuals are pooled as it increases probability that the observed allele ratio will be concordantly to segregation rules 50%: 50%,
3. If possible, a cross of distant populations is recommended (Spanish and Czech populations were used in the present work as the mother and father samples, respectively) as it increases the probability of allele variability,
4. Users are encouraged to use long pair-end reads for sequencing in order to lower the number of reads that cannot be mapped uniquely,
5. If possible, strand-specific RNA-Seq protocol should be used preferably (with Trinity parameters adjusted accordingly)

For this project, we designed filtering rules for each segregation pattern and implemented rules for pattern A (Table 1). According to these rules, only contigs representing high quality sex-linked markers are preserved.

**Table 1:** **List of filtering rules used for identification of sex-linked contigs; filtering rules for identification of X-linked genes following segregation pattern A**

**Mother must be homozygous (X** ^**1**^ **X** ^**1**^ **).**	Especially is checked whether mother contains any father variants (X^2^). Limited tolerance of these variants is described in Table 3.
**Sons must be same as mother (X** ^**1**^ **).**	First, we have to exclude male variants (variants shared between father and son but absent in daughter). Then, sons must contain only X^1^ variants of mother.
**Father must differ from mother.**	Father must have at least one SNP (X^2^) in which it differs from mother.
**Daughters must have mother and father alleles (X** ^**1**^ **X** ^**2**^ **).**	Only heterozygous daughter contigs are taken into account. These daughter variants must be subset of father variants.
**Daughters and sons cannot have any intersection.**	Sons are tested if they contain daughter variants with tolerance described in Table 3. Similarly, daughters are tested for presence of son variants.
**Sufficient coverage.**	Average depth per nucleotide must be at least 0.75 (=in contig of length 100, there must be at least 75 nucleotides present/mapped).

The pipeline is accessible under the list of published Galaxy workflows on the webpage: usegalaxy.org/workflow/list_published. It incorporates custom tools, such as LINKYX_X, LINKYX_Y and LINKYX_mpileup for identification of X-linked genes, identification of Y-linked genes and custom mpileup, respectively. All these tools can be installed from Galaxy Tool Shed to any Galaxy instance [8-10]. In our experience, a typical dataset with a total of 25 million reads requires a computation time in the range of 18–36 hours, with most of the time dedicated to de-novo assembly. The project home page at github.com/biomonika/linkyx/ describes how to install the tools and built-in wrappers of the tools should install all of the dependencies automatically.

### Identification of X-linked genes

In order to obtain transcripts of putative X-linked genes, all mother reads were assembled into contigs with Trinity assembler [11]. These contigs therefore represented the reference. This is a crucial step of the procedure since in previous works [12,13], the reference was derived from all individuals. Including all sequences from all individuals for building reference contigs necessarily leads to the creation of an artificial individual. All polymorphisms then need to be compared to this artificial individual and subsequently between each other (as the reference will just contain the most abundant variant with no biological meaning). Also, some contigs will come from male-specific sequences only and the method will fail when diverged X and Y homologs are not assembled together into one contig. Therefore, building reference contigs exclusively from the mother’s dataset significantly aids the gene detection process.

In the next step, reads from all family members are mapped back onto the contigs with BWA (default parameters) [14]. Four SAM/BAM files containing alignment of reads are created with Samtools and sorted [15]. Duplicate reads, which may result from artificial PCR amplification step during sequencing procedure, are removed. Three software solutions have been tested for this procedure - Picard’s MarkDuplicates function (http://broadinstitute.github.io/picard/), SAMTools’ rmdup and Fastx collapse (http://hannonlab.cshl.edu/fastx_toolkit/). Since Fastx collapse cannot produce FASTQ files and MarkDuplicates repeatedly reported biggest number of properly paired reads and lowest number of singletons for our data, it was chosen for further use in the pipeline.

Subsequently, variants were called. SAMtools mpileup was set to -uf and bcftools view was used with following parameters: “-p 0.85 -cgv -”. Resulting VCF files contain SNPs, indels and short structural rearrangements. In principle, they contain all information with regard to how a sample differs from the reference sequence. SAMtools parameters have been chosen by rule of thumb after extensive testing. It must be taken into account that reporting many variants that will contain sequencing errors or misalignments can lead to excluding a true X-linked gene for technical reasons, reporting fewer variants may lead to reporting autosomal genes that break one of our filtering criteria with these fraudulences not being recognized. The selected criteria should thus represent a balance between both the above-mentioned problems – the first of which raises the level of false negatives and the second which raises the level of false positives.

Beside variants generated by SAMtools, the program itself checks for variants that could not be reported by SAMtools and still should be forbidden at certain positions. For example, if there are 30 reads covering a certain position, 28 of them reporting adenin and 2 reporting cytosin, samtools may not report the 2 cytosins. However, if according to filtering rules cytosins are forbidden at that position, the program excludes the whole contig from further computation (for instance, when the mother contains few reads carrying father X^2^ allele). In this way, the initial dataset (typically thousands of contigs) is filtered to contain only candidate transcriptionally active sex-linked markers.

**Figure 3:**
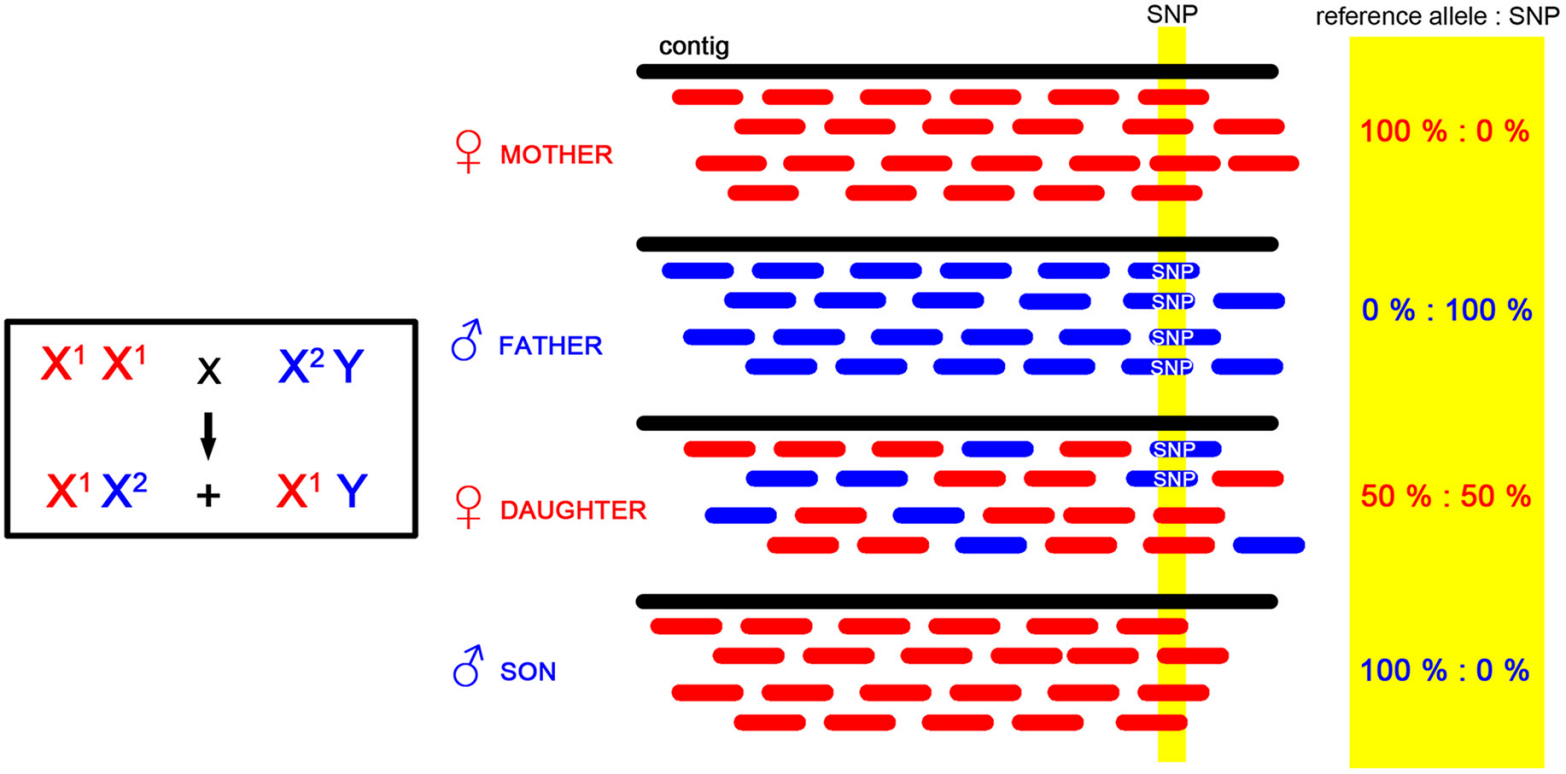
**Schematic representation of identification of X-linked genes exhibiting the A pattern.** Mother is homozygote (X^1^X^1^), father has a different variant (X^2^). Approximately half of the daughter’s reads follow the father’s variant (SNP) while sons inherit mother’s variant. Variants specific only for males suggesting the Y origin are neglected.

Custom bash and perl scripts were written for the filtering step with rules described in Tables 1, 2 and 3. Genes exhibiting pattern A are filtered based on 6 rules described in Table 1 and the principle of filtering is visualized in Figure 3.

**Table 2:** **List of filtering rules used for identification of sex-linked contigs; filtering rules for identification of Y-linked genes**

**Minimal and maximal number of reads.**	Each male must have at least 15 reads mapped, each female can have at most 3 reads mapped.
**Fraction of female and male reads.**	female reads/(female reads + male reads) < 0.03.
**Male reads must be more abundant than female reads.**	mother reads < father reads;
	mother reads < son reads;
	daughter reads < father reads;
	daughter reads < son reads

**Table 3:** **List of filtering rules used for identification of sex-linked contigs; threshold for variant tolerance used in Table** 1

**Depth in number of reads:**	**Tolerated depth of alternative allele (SNP) in number of reads:**
**0-4**	0
**5-24**	1
**> = 25**	<=4 %

For every set of genes, the following information is provided: contig name, sequence, isoforms listed (in case they exist). The list of genes is sorted. For X-linked genes, their list is sorted by the number of uniquely mapped reads for each family member, following the expectation that contigs with the least number of such reads are the easiest to be confirmed as truly X-linked (no isoforms or duplicated genes).

### Identification of Y-linked genes

**Figure 4:**
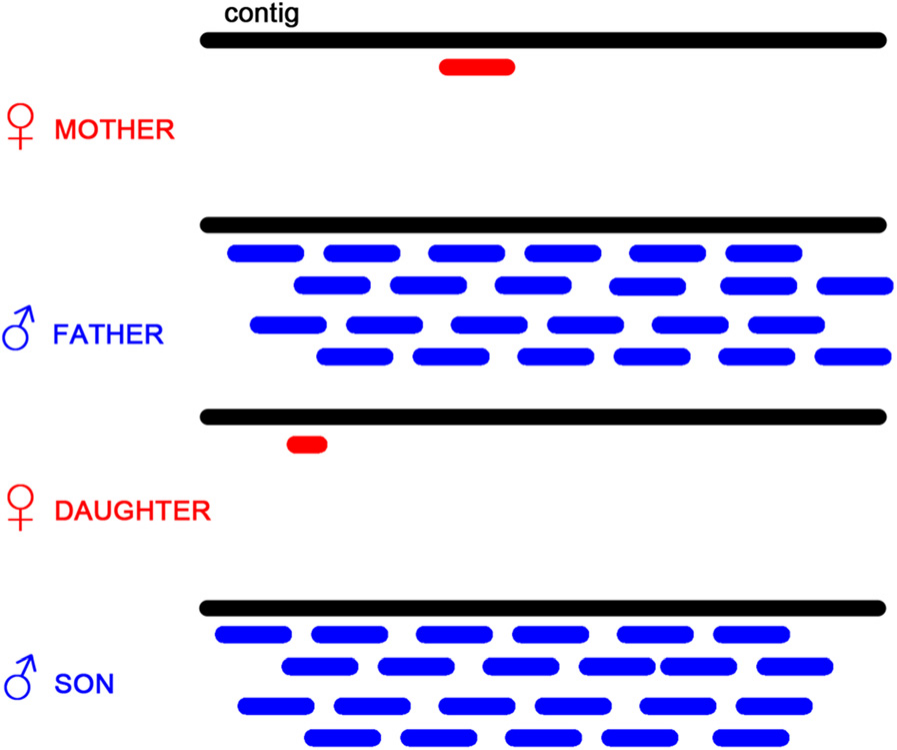
**Schematic representation of identification of Y-linked genes.** Reference contigs are assembled from male reads only. Then, female reads do not map to contigs of Y-chromosome origin while male reads do. Short fragments in red color visualized on the figure represent limited tolerance of mapping/assemble errors.

In order to obtain transcripts of putative Y-linked genes, all male reads were assembled into contigs with Trinity assembler. All reads are then mapped back, sorted and deduplicated in a manner that is similar to the previous case. If the contigs come from the Y chromosome or are expressed specifically in males, then the female reads do not map to those contigs, while male reads do (Figure 4). Four BAM files (mother, father, male and female offspring) are therefore filtered according to designed rules described in Table 2. Before applying these rules, read numbers are normalized to the number of mother reads.

To test that proposed filters sufficiently separated Y-linked contigs from contigs present in both sexes, all reads were mapped again on the male reference contigs with highly relaxed conditions represented by BWA parameter aln -n 0.001. With these conditions, 51.63% of reads were mapped instead of 49.12%, which confirmed reliability of chosen parameters and showed that identified contigs were either very divergent Y copies or standalone genes. Again, putative genes are sorted from the most relevant ones. The key for sorting is coverage, with the first contig covered by the biggest number of reads.

## Results

### PCR confirmation of sex chromosome-linkage of identified genes

**Figure 5:**
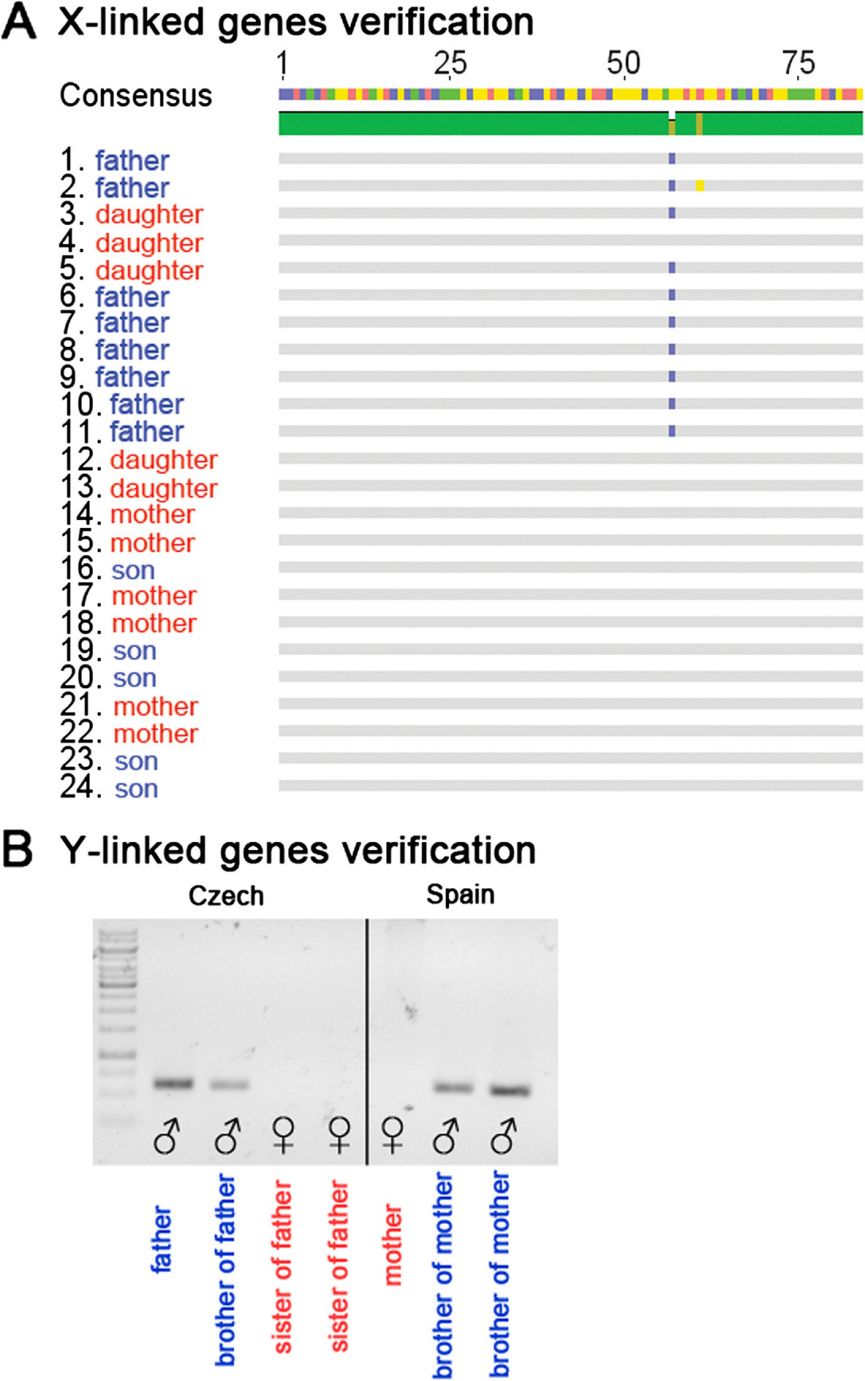
**Experimental laboratory verification of sex-linked contigs. A)** PCR products of candidate X-linked contigs were sequenced and clustered. Father and daughter variants cluster together, which confirms X-linkage of a selected gene. Note that SNP marked in blue color is shared only among sequences of father and daughters. Another SNP in yellow represents sequencing error. **B)** PCR products of candidate Y-linked contigs. For every contig/gene genomic DNA of 7 individuals (4 males, 3 females) was used as a template: father (Reckovice, CZ), brother of father (Reckovice, CZ), two sisters of father (Reckovice, CZ), mother (Almería, Spain), two brothers of mother (Almería, Spain). Product is present only in male individuals.

45 candidate X-linked genes and 24 candidate Y-linked genes have been obtained by the pipeline presented here. From these, 11 X-linked genes and 8 Y-linked genes were randomly chosen for wet-lab confirmation. First, PCR was performed for the 11 candidate X-linked genes, PCR products were cloned and sequenced. Manual inspection of these sequences (clustering of sequenced sequences, which showed that father and daughter variants clustered together) confirmed their X-linkage (Figure 5A). In order to verify the Y-linkage of predicted genes, PCR was performed using DNA from male and female plants of parental generation. A majority of genes (7 of 8 genes) showed a prominent male PCR product suggesting their Y origin (Figure 5B, Additional file 1: Table S1). In the case of one contig, PCR products could be seen in both male and female samples suggesting that this contig might be present in both sexes, while manifesting a sex-dependent expression.

In order to exclude the possibility that the patterns of 7/8 contigs were caused by difference between the Spanish (mother) and the Czech (father) populations, new individuals were included from both populations. This step confirmed that 6 out of 8 contigs were truly of Y chromosome origin. In one case, PCR products were missing in the Spanish population which suggests that this population has different variants on primer-binding sites. Taken together, 6/8 genes were reliably confirmed and none were certainly refuted.

For X-linked verification, we used the DNA from father, mother and pooled male and female progeny. We sequenced 20–40 sequences for each contig after amplification. After alignment, contigs where father allele was passed only to daughters (not present neither in mother nor in son sequences) were considered X-linked. We were able to reliably confirm that 4 contigs were truly X-linked. In 6 contigs, a conclusion could not be made due to the low coverage and the possibility of not sampling all alleles. One contig represented false positive result and was probably of autosomal origin.

## Discussion

Recently, Chibalina et al. [13] used an RNA-Seq based approach for identification of sex-linked genes in *Silene latifolia*, another dioecious plant with XY sex chromosomes. They used two different crosses and subsequent sequencing of all members of the family (mother, father, daughters, and sons) for identification of marker chromosome linkage. The same approach was used by Hough et al. [16] to identify sex-linked genes in the dioecious plant *R. hastatulus* with a neo-Y sex chromosome system. They used transcriptomes from parents and F_1_ progeny from two within-population crosses, one from a population with XY males and one from a population with XY_1_Y_2_ males. Similar strategy was used by Muyle et al. [12] who analysed a 10-generations inbred line of *Silene latifolia* for determination of sex linkage of genes using RNA-Seq. Three males and three females were pooled and massively sequenced. Subsequently, contigs representing consensus sequence for all individuals were assembled together. In such an inbred line, a very low heterozygosity was expected and thus the contigs were searched for a pattern where all males showed the heterozygous XY pattern, and all females presented the homozygous XX pattern. It was expected that in males, reads of the X chromosome origin would be the same as those in females, whereas reads from the Y chromosome would exhibit a new variant. All three reports predicted hundreds of new X- or Y-linked genes. However, we assume that some predicted genes could be false positives, since segregation of any SNP according to expected pattern was sufficient for considering contigs as X/Y-chromosome linked. In contrast, in our approach all SNPs in analyzed expressed markers exhibit expected segregation. Therefore, the number of predicted genes is lower but reliability is higher. For this reason, genes predicted by our tool are more suitable as an initial dataset for isolation of Y- and X-linked BAC clones and physical mapping of sex chromosomes. It should be noted that our approach would not identify sex-linked genes that are not recombinationally trapped (i.e. pseudoautosomal) or are recent additions to non-recombining regions. We conclude that this approach together with experimental laboratory verification represents a reliable tool for obtaining sex-linked markers for non-model species.

## Conclusions

In this work, we developed a bioinformatics approach for detection of sex-linked genes using RNA-Seq data. Such an approach could be used for identification of both Y (and W) chromosome-linked or X (Z) chromosome-linked genes in animal and plant species. No bioinformatics skills are needed for users of our pipeline since FASTQ files (generated directly by sequencing machines) are the only input for data processing. The tool is freely accessible to the scientific community via github.com/biomonika/linkyx/. As a proof of concept, RNA-Seq data were used in combination with a cross of parents and F1 male and female offspring in *R. acetosa*. After de novo assembly of Illumina based data we identified X- or Y- linked patterns of segregation based on nucleotide polymorphism analysis. For validation of our prediction, we selected Y-specific EST for PCR experiments that proved the reliability of our approach for high throughput identification of sex-linked markers. Moreover, we showed for the first time that *R. acetosa* heterochromatic Y chromosomes contain transcriptionally active genes. Prior to the present work, only repetitive DNA were shown to be gathered on the both Y chromosomes in this species [17].

## Availability and requirements

- **Project name:** Fully automated pipeline for detection of sex linked genes using RNA-Seq data.
- **Project home page:**github.com/biomonika/linkyx/.
- **Operating system(s):** UNIX-based, primarily tested on Debian.
- **Programming language:** bash, Perl, Python.
- **Other requirements:** Java 1.6.
- **License:** Academic Free License 3.0.
- **Any restrictions to use by non-academics:** no.

## Additional file

Additional file 1: Table S1. X-linked and Y-linked primers and sequences used for verification.

## References

[1] Vyskot B, Hobza R (2004). Gender in plants: sex chromosomes are emerging from the fog. Trends Genet.

[2] Hobza R, Vyskot B (2007). Laser microdissection-based analysis of plant sex chromosomes. Methods Cell Biol.

[3] Hobza R, Hrusakova P, Safar J, Bartos J, Janousek B, Zluvova J (2006). MK17, a specific marker closely linked to the gynoecium suppression region on the Y chromosome in Silene latifolia. Theor Appl Genet.

[4] Moore RC, Kozyreva O, Lebel-Hardenack S, Siroky J, Hobza R, Vyskot B (2003). Genetic and functional analysis of DD44, a sex-linked gene from the dioecious plant Silene latifolia, provides clues to early events in sex chromosome evolution. Genetics.

[5] Soneson C, Delorenzi M (2013). A comparison of methods for differential expression analysis of RNA-seq data. BMC Bioinformatics.

[6] Ainsworth C (1994). Isolation of RNA from floral tissue of Rumex acetosa (Sorrel). Plant Mol Biol Report.

[7] Błocka-Wandas M, Sliwinska E, Grabowska-Joachimiak A, Musial K, Joachimiak AJ (2007). Male gametophyte development and two different DNA classes of pollen grains in Rumex acetosa L., a plant with an XX/XY1Y2 sex chromosome system and a female-biased sex ratio. Sex Plant Reprod.

[8] Goecks J, Nekrutenko A, Taylor J (2010). Galaxy: a comprehensive approach for supporting accessible, reproducible, and transparent computational research in the life sciences. Genome Biol.

[9] Blankenberg D, Von Kuster G, Coraor N, Ananda G, Lazarus R, Mangan M (2010). Galaxy: a web-based genome analysis tool for experimentalists. Current Protoc Mol Biol..

[10] Giardine B, Riemer C, Hardison RC, Burhans R, Elnitski L, Shah P (2005). Galaxy: a platform for interactive large-scale genome analysis. Genome Res.

[11] Grabherr MG, Haas BJ, Yassour M, Levin JZ, Thompson DA, Amit I (2011). Full-length transcriptome assembly from RNA-Seq data without a reference genome. Nat Biotechnol.

[12] Muyle A, Zemp N, Deschamps C, Mousset S, Widmer A, Marais GAB (2012). Rapid De Novo Evolution of X Chromosome Dosage Compensation in Silene latifolia, a Plant with Young Sex Chromosomes. PLOS Biol.

[13] Chibalina M, Filatov D (2011). Plant Y chromosome degeneration is retarded by haploid purifying selection. Curr Biol.

[14] Li H, Durbin R (2009). Fast and accurate short read alignment with Burrows-Wheeler transform. Bioinformatics.

[15] Li H, Handsaker B, Wysoker A, Fennell T, Ruan J, Homer N (2009). The Sequence Alignment/Map format and SAMtools. Bioinformatics.

[16] Hough J, Hollister JD, Wang W, Barrett SC, Wright SI (2014). Genetic degeneration of old and young Y chromosomes in the flowering plant Rumex hastatulus. Proc Natl Acad Sci U S A.

[17] Steflova P, Tokan V, Vogel I, Lexa M, Macas J, Novak P (2013). Contrasting patterns of transposable element and satellite distribution on sex chromosomes (XY1Y2) in the dioecious plant Rumex acetosa. Genome Biol Evol.

